# Supplementary material for: Toxicological Effects of Naturally Occurring Endocrine Disruptors on Various Human Health Targets: A Rapid Review
Source: Toxics. 2024 Mar 29;12(4):256. doi: 10.3390/toxics12040256 (PMC11054122; doi:10.3390/toxics12040256)
Supplement: Supplementary file 1 [file toxics-12-00256-s001.zip › toxics-2869703-supplementary.pdf]

Search: (((("ENDOCRINE DISRUPTORS"[PHARMACOLOGICAL ACTION]) OR ("ENDOCRINE DISRUPTORS"[MESH]) OR ("ENDOCRINE DISRUPTOR"[tiab:~5] OR "ENDOCRINE DISRUPTORS"[tiab:~5] OR "ENDOCRINE DISRUPTION"[tiab:~5] OR "ENDOCRINE DISRUPTING"[tiab:~5] OR "ENDOCRINE DISRUPTER"[tiab:~5] OR "ENDOCRINE DISRUPTERS"[tiab:~5] OR "ENDOCRINE DISRUPT*")) AND (("8-PRENYLNARINGENIN" [SUPPLEMENTARY CONCEPT] OR BISPHENOL F [SUPPLEMENTARY CONCEPT] OR ANDROGENS [PHARMACOLOGICAL ACTION] OR "PHYTOESTROGENS" [PHARMACOLOGICAL ACTION] OR "COUMESTAN" [SUPPLEMENTARY CONCEPT] OR "DAIDZEIN" [SUPPLEMENTARY CONCEPT] OR "GALLOCATECHOL" [SUPPLEMENTARY CONCEPT] OR "PEONIFLORIN" [SUPPLEMENTARY CONCEPT] OR "PREGNA-4,17-DIENE-3,16-DIONE" [SUPPLEMENTARY CONCEPT] OR "ALTERNARIOL" [SUPPLEMENTARY CONCEPT]) OR ("COUMARINS"[MESH] OR "MYCOTOXINS"[MESH] OR "ANTHRAQUINONES"[MESH] OR "CURCUMIN "[MESH] OR "GOSSYPOL"[MESH] OR "PHYTOESTROGENS"[MESH TERMS] OR "FLAVONOIDS"[MESH TERMS] OR "LIGNANS"[MESH TERMS] OR "STILBENES"[MESH TERMS] OR "PHYTOSTEROLS"[MESH TERMS] OR "OILS, VOLATILE"[MESH TERMS] OR "EMODIN"[MESH TERMS] OR "MELALEUCA"[MESH TERMS] OR "SOYBEANS"[MESH TERMS] OR "SOY FOODS"[MESH TERMS] OR "ZEARALENONE"[MESH TERMS]) OR ("8-prenylnaringenin*"[tiab] OR "BISPHENOL F"[tiab] OR ANDROGENS[tiab] OR COUMESTROL*[tiab] OR COUMARINS*[tiab] OR ISOCOUMARINS*[tiab] OR OCHRATOXIN*[tiab] OR MYCOTOXIN*[tiab] OR FLAVON*[tiab] OR APIGENIN[TIAB] OR KAEMPFEROLS*[TIAB] OR ANTHRAQUINONE*[TIAB] OR ZERANOL*[TIAB] OR CURCUMIN[TIAB] OR GOSSYPOL[TIAB] OR PHYTOESTROGEN*[tiab] OR ISOFLAVONE*[tiab] OR GENISTEIN[tiab] OR LUTEOLIN[tiab] OR QUERCETIN[tiab] OR "ESSENTIAL OIL"[tiab] OR "ESSENTIAL OILS"[tiab] OR "TEA TREE OIL"[tiab] OR DAIDZEIN[tiab] OR EPIGALLOCATECHIN*[tiab] OR PAEONIFLORIN[tiab] OR PEONIFLORIN[tiab] OR GUGGULSTERONE[tiab] OR EMODIN[tiab] OR MELALEUCA[tiab] OR SOYBEAN*[tiab] OR SOY[tiab] OR ZEARALENONE[tiab] OR ALTERNARIOL[tiab] OR RESVERATROL[tiab] OR COUMESTAN*[tiab]))) AND ((humans[Filter]) AND (english[Filter]) AND (2019:2023[pdat]))) Filters: Humans, English, from 2019 - 2023

Sent On: Mon Sep 25 10:17:06 2023

Figure S1. Complete "Full string" used for research strategy, in order to interrogate the PubMed database and to found the eligible papers.
